# Retraction note: Decrease expression and clinicopathological significance of miR-148a with poor survival in hepatocellular carcinoma tissues

**DOI:** 10.1186/s13000-016-0561-8

**Published:** 2016-11-02

**Authors:** Hossein Ajdarkosh, Masoomeh Dadpay, Emad Yahaghi, Elham Rostami Pirzaman, Amir Farshid Fayyaz, Ebrahim Khodaverdi Darian, Aram Mokarizadeh

**Affiliations:** 1 Gastrointestinal and Liver Disease Research Center (GILDRC), Firoozgar Hospital, Iran University of Medical Sciences, Tehran, Iran; 2 Department of Pathology, Imam Reza Hospital, AJA University of Medical Sciences, Tehran, Iran; 3 Baqiyatallah University of Medical Sciences, Tehran, Iran; 4 Department of Biotechnology and Nanotechnology, Zanjan University of Medical Sciences, Zanjan, Iran; 5 Department of Legal Medicine, AJA University of Medical Sciences, Tehran, Iran; 6 Young Researchers and Elite Club, Karaj Branch, Islamic Azad University, Karaj, Iran; 7 Cellular & Molecular Research Center, Kurdistan University of Medical Sciences, Sanandaj, Iran

## Retraction

The Editor-in-Chief and Publisher have retracted this article [1] because the scientific integrity of the content cannot be guaranteed. An investigation by the Publisher found it to be one of a group of articles we have identified as showing evidence suggestive of attempts to subvert the peer review and publication system to inappropriately obtain or allocate authorship. This article showed evidence of plagiarism (most notably from the articles cited [2, 3]) and authorship manipulation.
